# The Cambridge Structural Database and structural dynamics

**DOI:** 10.1063/4.0000244

**Published:** 2024-03-18

**Authors:** Hans-Beat Bürgi

**Affiliations:** Department of Chemistry, Biochemistry and Pharmaceutical Sciences, University of Berne, Freiestr. 3, CH-3012 Bern, Switzerland

## Abstract

With the availability of the computer readable information in the Cambridge Structural Database (CSD), wide ranging, largely automated comparisons of fragment, molecular, and crystal structures have become possible. They show that the distributions of interatomic distances, angles, and torsion angles for a given structural fragment occurring in different environments are highly correlated among themselves and with other observables such as spectroscopic signals, reaction and activation energies. The correlations often extend continuously over large ranges of parameter values. They are reminiscent of bond breaking and forming reactions, polyhedral rearrangements, and conformational changes. They map—qualitatively—the regions of the structural parameter space in which molecular dynamics take place, namely, the low energy regions of the respective (free) energy surfaces. The extension and continuous nature of the correlations provides an organizing principle of large groups of structural data and suggests a reconsideration of traditional definitions and descriptions of bonds, “nonbonded” and “noncovalent” interactions in terms of Lewis acids interacting with Lewis bases. These aspects are illustrated with selected examples of historic importance and with some later developments. It seems that the amount of information in the CSD (and other structural databases) and the knowledge on the nature of, and the correlations within, this body of information should allow one—in the near future—to make credible interpolations and possibly predictions of structures and their properties with machine learning methods.

The reader may wonder about the combination of topics in the title of this contribution: structural dynamics and the Cambridge Structural Database (CSD) (Groom *et al.,* 2016). The latter is a comprehensive compendium of more than 1 250 000 crystal structures, essentially tables of atomic positions (and some atomic displacement parameters), which represent averages over all unit cells of an investigated crystal and the time needed to measure it. No obvious relationship to the essence of chemistry, namely, structural dynamics of more or less flexible molecules forming and breaking bonds. As I hope to illustrate in this review, the connection is one of the consequences of the visionary motivation at the origin of the CSD: “We (Olga Kennard and John Desmond Bernal) had a passionate belief that the collective use of data would lead to the discovery of new knowledge, which transcends the results of individual experiments.” (“*Celebrating Dr Olga Kennard OBE FRS, Founder of the Cambridge Structural Database, 1924–2023, Obituary,*” https://www.ccdc.cam.ac.uk/discover/news/celebrating-dr-olga-kennard-1924-2023/).

This contribution presents some of this knowledge; in particular, results related to chemical transformations. They have been discovered by making “collective use of data,” i.e., by viewing entire families of crystal or molecular structures from a common point of view (structure correlation). The members of these families are sometimes very similar, differing only in a single experimental parameter (e.g. temperature); sometimes they are widely different overlapping only in a common structural fragment. The concept used to establish connections between “static” crystal structures and chemical dynamics is illustrated with a limited number of examples chosen for their historic importance or their role as a basis for further work, especially correlations with spectroscopic, thermodynamic, and other physical observables. Several reviews, book articles, and the references therein provide detailed information (Auf Der Heyde, 1994; Bürgi, 1975; 1992; 1998; 2002; Bürgi and Dunitz, 1983; 1994; Dunitz, 1979; 1995; Ferretti *et al.,* 1996; Grabowski, 2020a; Klebe, 1990; 1994; and Orpen, 2002).

## I. STATISTICAL METHODS, CORRELATIONS, AND DATA RETRIEVAL

An almost trivial usage of large numerical databases is to do statistics. Chapter 9 of Volume C of the International Tables of Crystallography characterizes typical interatomic distances for metals, alloys, inorganic, organic, and organometallic compounds. The distances for a wide range of chemical bonds have been retrieved from the CSD and tabulated in terms of their mean, median, standard deviation, upper, and lower quartiles (Prince, 2006). With the help of these tables, unusual interatomic distances in new structure determinations are easily identified. Such outliers could hint at special chemical factors or at a potential experimental error. Statistical comparisons for interatomic distances, bond, and torsion angles in tailor-made structural fragments of interest are also available in the MOGUL geometry check tool available independently (https://www.ccdc.cam.ac.uk/solutions/software/mogul/) or in the MERCURY program (https://www.ccdc.cam.ac.uk/solutions/software/mercury/) of the CSD software.

N-atomic molecular or crystal structure fragments are characterized by 3N-6 or more interatomic distances, angles, and torsion angles, which define a multidimensional distribution. Such distributions may be uni- or multi-modal. Uni-modal distributions are characterized not only by their mean values but also by covariances between their parameters. Large covariances indicate correlated changes of the covariant parameters, which can be scrutinized for chemical explanation. Multi-modal distribution may be analyzed for local clusters with cluster analysis methods (https://en.wikipedia.org/wiki/Cluster_analysis) and clusters characterized like unimodal distributions. Often, not all of the structural parameters (or combinations thereof) are of interest or show significant variations. Principal component analysis reduces the dimensionality of a distribution and allows to extract the important correlations (https://en.wikipedia.org/wiki/Principal_component_analysis). Only those (correlated) structural changes are considered that are associated with principal components larger than a user defined threshold. A tutorial summarizes the use of these techniques in structure correlation studies (Auf Der Heyde, 1990).

**FIG. 1.**
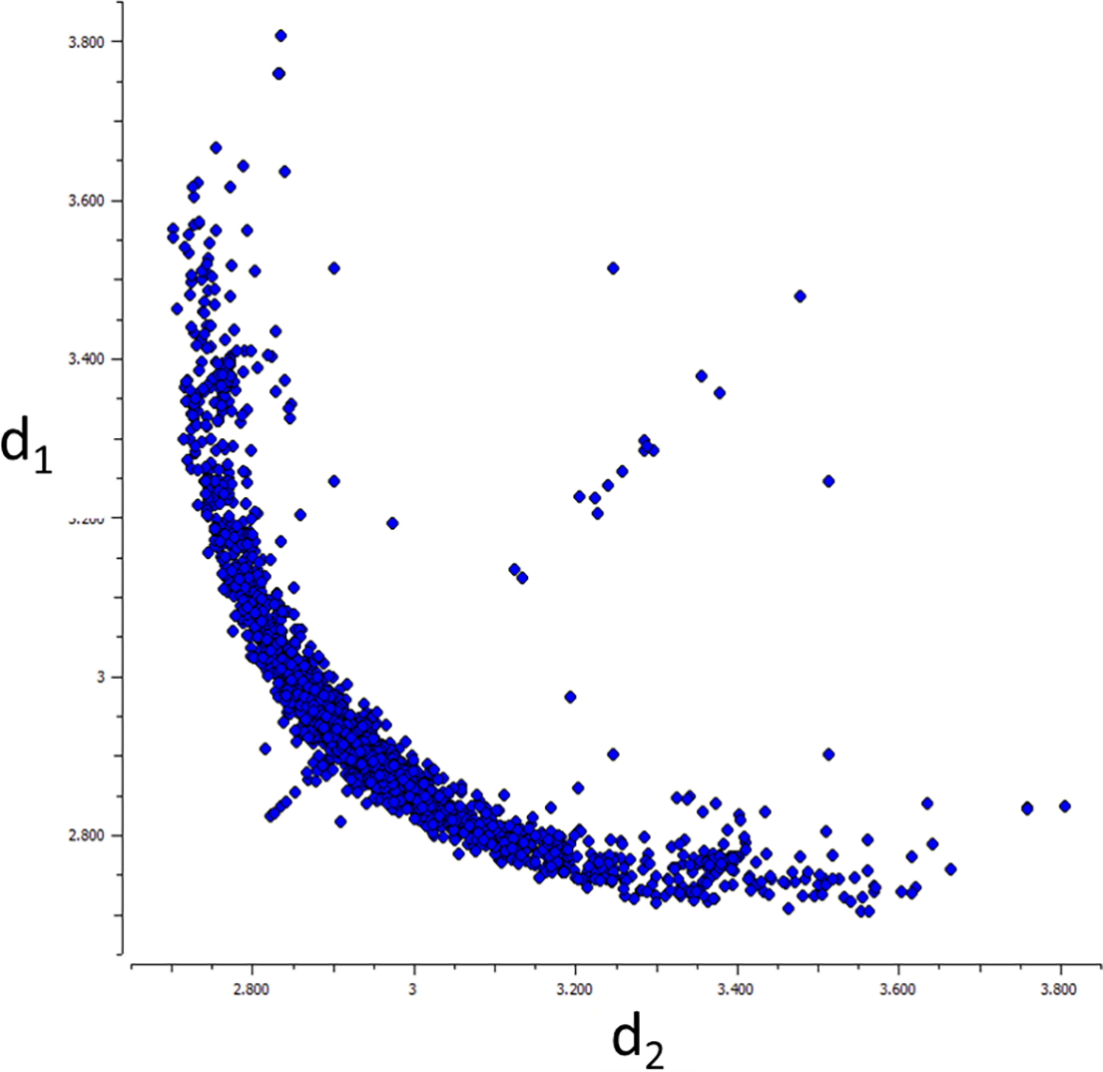
Scatterplot of d_1_(I1.I2) vs. d_2_(I2.I3) in I_3_–fragments with an I1.I2.I3 angle in the range 165-180° (CSD, April 2023, 1004 hits, mirror symmetry about d_1_ = d_2_ imposed, 1703 symmetry independent data points, R ≤ 0.05, no disorder, no errors, no powders).

An early, simple example of correlated structural parameters was provided by Bent (1968); he plotted the two I.I distances for eight I_3_^−^-fragments, one against the other. The data were taken from eight different crystal structures differing mainly in the positive counterion. The distribution of datapoints was not random but followed a hyperbola-like curve. An updated version of Bent's plot is given in Fig. 1. The scatterplot shows three obvious features: most points by far are found near Bent's original hyperbola-like curve; a smaller group of points are found on the diagonal d_1_ = d_2_ (∼1%). A third group of points is scattered more or less randomly (∼1%).

**FIG. 2.**
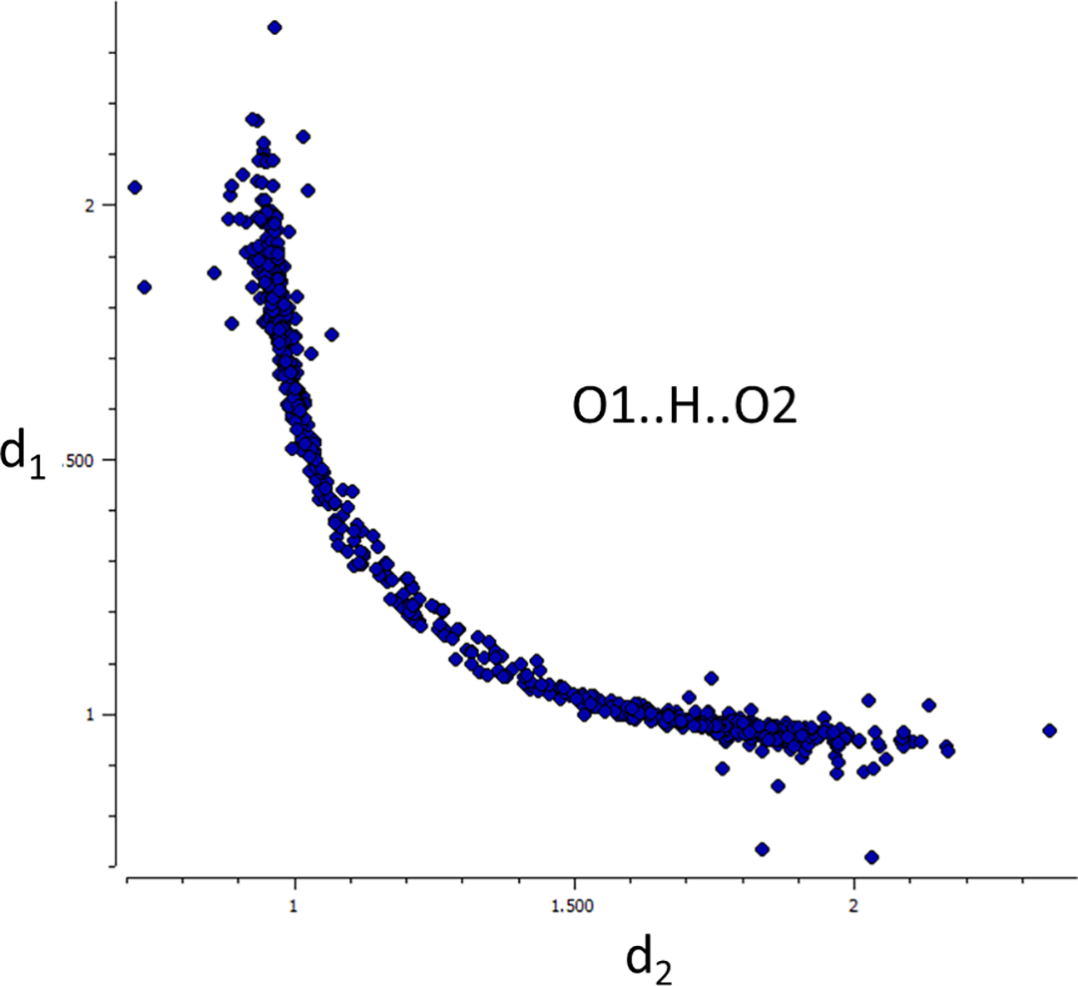
Scatterplot of d_1_(O1.H) vs. d_2_(H.O2) in O1.H.O2 fragments with an O1.H.O2 angle in the range 165–180° (CSD, April 2023, neutron diffraction, 167 hits, mirror symmetry about d_1_ = d_2_ imposed, ∼425 symmetry independent data points, R ≤ 0.05, no disorder, no errors, no powders).

Bent's data and other early correlations have been compiled largely by traditional literature search in the libraries or—somewhat easier—by consulting the hard copy bibliography of organic and organometallic crystal structures “Molecular Structure and Dimensions,” which have been compiled since 1970 by the Cambridge Crystallographic Data Center (CCDC; Allen *et al.,* 1982). The 14 published volumes cover the period 1935–1982. Toward the end of this period, the compiled information, including numerical data, became available as computer searchable files. In Switzerland, on-line access to the CSD was established in 1977.

The idea of correlating structural parameters characterizing the same fragment structure in widely different environments together with the on-line availability of structural databases like the CSD established the field of “Structure Correlation,” a flourishing crystallographic research topic during the last quarter of the 20th century.

## II. INTERPRETATION OF CORRELATIONS BETWEEN STRUCTURAL PARAMETERS

Correlated changes of structural parameters identified by qualitative visual, or quantitative statistical methods and extending over large ranges of structural parameters (e.g., interatomic distances, Figs. 1 and 2) call for an interpretation in chemical terms. A fruitful way to discuss such correlations is to relate them to making and breaking bonds, to changing conformations and to distorting structural fragments in general. This is exactly how Bent (1968) interpreted his correlation consisting of eight points: “The hyperbolic-like curve may be presumed to show, approximately, the changes that occur in the distances between nearest neighbors in the linear exchange reaction I_1_ + I_2_I_3_ = I_1_I_2_ + I_3_ …”! Although Bent makes no explicit mention of energy or dynamics, the association with the breaking and making of I-I bonds along a well-defined reaction path involving changes in two parameters, the two I.I distances, is unmistakable. A visionary interpretation indeed, several hundredfold confirmed between 1968 and 2023! (Fig. 1) The updated plot also raises questions: what about the points that are not on the hyperbola-like curve? Are they indicative of unusual structural and chemical bonding situations or do they represent cases of erroneous symmetry assignment or unresolved disorder? Such questions can and must be asked given a background of strong correlations encompassing the vast majority of a class of fragment structures.

**FIG. 3.**
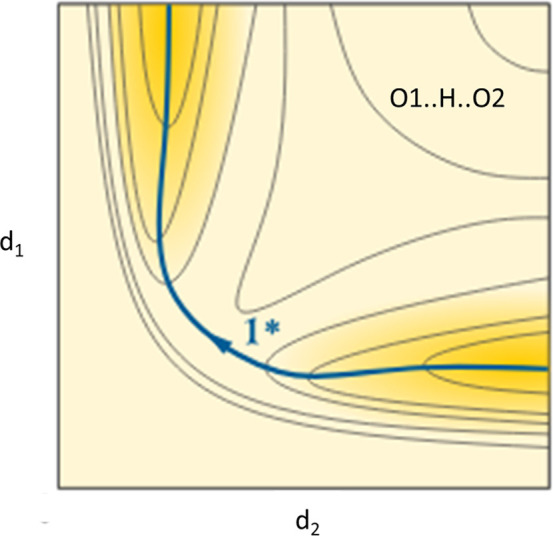
Qualitative energy surface for an O1.H.O2 fragment as a function of d_1_(O1.H) vs. d_2_(H.O2). (Adapted from LibreTexts Chemistry, chapter 30.10, Fig. 30.10.6.A).

### A. Principle of structural correlation

However, what is the physical origin of these correlations? Comparison of Figs. 2 and 3 suggests an answer. The former shows a scatterplot of the two O.H distances in O.H.O fragments. In analogy to I exchange in I_3_^–^, the correlation can be interpreted to picture the pathway for proton transfer. Figure 3 shows a qualitative rendering of the potential energy surface (PES) for a proton transfer reaction as calculated with quantum chemical methods for an (H_2_O.H.OH_2_)^+^, (HO.H.OH)^–^ or similar aggregate containing an O.H.O fragment. The blue line represents the curve of steepest descent (or Minimum Energy Pathway, MEP) from the transition state 1^*^ into the valleys O1–H…O2 and O1…H–O2. The similarity between the correlation curve in Fig. 2 and the MEP in Fig. 3 is obvious. Analogous observations have led Murray-Rust *et al.* (1975) to formulate the “Principle of Structural Correlation”: “We have assumed as a working hypothesis that if a correlation can be found between two or more independent parameters describing the structure of a given structural fragment in a variety of environments, then the correlation function maps a minimum energy path in the corresponding parameter space.” The relative positions of fragments in a distribution are determined by the differences in their environments, which can be considered as mutual perturbations. For a given magnitude of the perturbation, the shift of a data point will be largest in directions with a small increase in the PES, i.e., generally along a reaction pathway. Examples relating structure, energy, and chemical transformations are scattered throughout this essay.

**FIG. 4.**
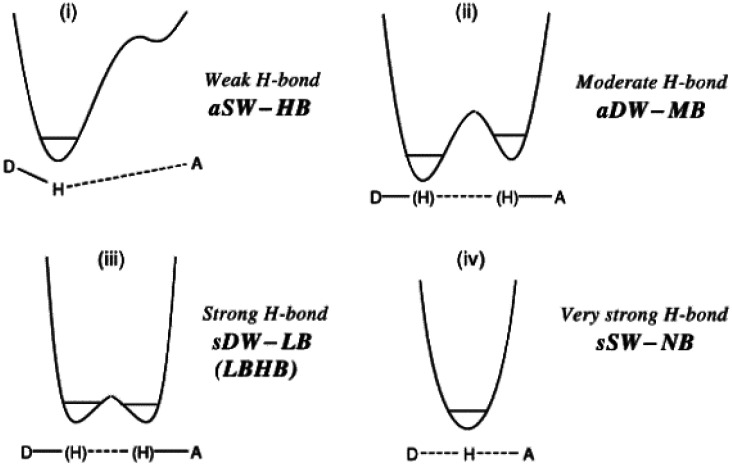
Structure-energy diagrams along the curved pathway of Fig. 3 for different hydrogen bonds. (Reproduced by permission from P. Gilli and G. Gilli, J. Mol. Struct. **972**, 2–10 (2010). Copyright 2010 by The Elsevier.)

### B. Correlations and bond orders

An empirical, extremely simple, and intuitive summary of the correlations shown in Figs. 1 and 2 derives from the idea that the sum n_1_ + n_2_ of the two bond orders describing the interactions of the central H or I with their terminal neighbors is a constant and that the bond orders can be expressed in terms of geometrical parameters

$$1\;={\;n}_{1}+{\;n}_{2}=\;\;\text{exp}\;\left\{\left(r_{1}-r_{10}\right)/c\right\}\;+\;\;\text{exp}\;\left\{\left(r_{2}-r_{20}\right)/c\right\}.$$

For 0.1.H.02 the r_i0_ are the lower limits of the O_i_.H distances; c is an empirical constant. The correlation displayed in Fig. 2 is adequately described by r_10_ = r_20_ = 0.91 Å and c = −0.43 Å (Bürgi, 1973). Analogous expressions have been formulated for several other correlations (Murray-Rust *et al.,* 1975; Bürgi, 1975).

### C. Dissecting the O.H.O correlation

Figure 3 hides the fact that the large range of correlated O.H distances data can be classified depending on the environment of the O.H.O fragment (Gilli and Gilli, 2010). Intermolecular hydrogen bonds in charged fragments, (D–H…A)^−^ or (D–H…A)^+^, tend to be strong and symmetric, i.e., located in the center of the distribution, especially if D = A (±charge assisted H-bonds; Gilli *et al.,* 1994). Intramolecular or intermolecular hydrogen bonds coupled through delocalized π-systems show a similar tendency (resonance assisted hydrogen bonds, e.g., the enol form of acetylacetone; Bertolasi *et al.*, 1997). Hydrogen bonds become increasingly asymmetric and weaker, i.e., move toward the tails of the distribution, as the difference ΔpK_a_ = pK_a_(D − H) − pK_a_(A − H) increases (“ordinary” hydrogen bonds; Gilli *et al.,* 2009). Asymmetric systems, primarily with D, A = O, N makeup the plethora of hydrogen bonds.

The generic reaction path-energy diagram in Fig. 3 has been adapted to the different types of hydrogen bonds (Fig. 4). For very strong H-bonds (short D…A distances), Gilli and Gilli (2010) postulate a parabolic energy dependence along the curved reaction pathway (Figs. 2 and 4) with a minimum for the symmetric structure. For strong, topologically symmetric H-bonds, the energy dependence takes the shape of a double minimum potential with an increasing barrier as the D…A distance increases. H shows static or dynamic disorder depending on temperature and the barrier height. For significant differences ΔpK_a_ with A more acidic than D, the double minimum potential becomes asymmetric along the reaction pathway with a global minimum for the D–H…A situation and a secondary minimum for D…H–A. For very large differences ΔpK_a,_ only the lower minimum remains, whereas the subsidiary minimum might reduce to a shoulder.

### D. Correlations with other observables

Hydrogen bond geometries correlate not only with ΔpK_a_'s but also with other observables, such as gas phase proton affinities and NMR chemical shifts (Bertolasi *et al.*, 1997; Limbach *et al.,* 2009). The correlations have been extended to a broad range of homonuclear, X.H^+^.X, and heteronuclear, X.H^+^.Y, fragments (Gilli *et al.,* 2009). Hydrogen bonds have also been studied by quantum chemical methods at many levels of sophistication. A discussion of these would be beyond the scope of this essay; Limbach *et al.* (2009) provide an example.

## III. NUCLEOPHILIC ADDITION/ELIMINATION REACTIONS

The reaction path for a nitrogen nucleophile approaching an electrophilic carbonyl group is probably the most widely known structure correlation, at least among organic chemists. In its original version, it shows an amine N approaching a RR′C=O group at an N…C=O angle between 100 and 110° (Bürgi-Dunitz angle). As the distance between N and the electrophilic C decreases the length of the C=O distance increases and the R_2_C=O group deforms toward a pyramid whose apex points toward N (Bürgi *et al.,* 1973). This correlation is based on a single, serendipitous observation of an unusually short “non-bonded” distance between an amine nitrogen and a carbonyl carbon atom in free base Methadone (2.91 Å vs a van der Waals distance of ∼3.3 Å; Rowland and Taylor, 1996); the correlation was established with five additional, published crystal structures of some pyrrolizidine alkaloids. The encyclopedic memory of Jack Dunitz and some classical literature search in the library provided the necessary information (Bürgi, 2022).

**FIG. 5.**
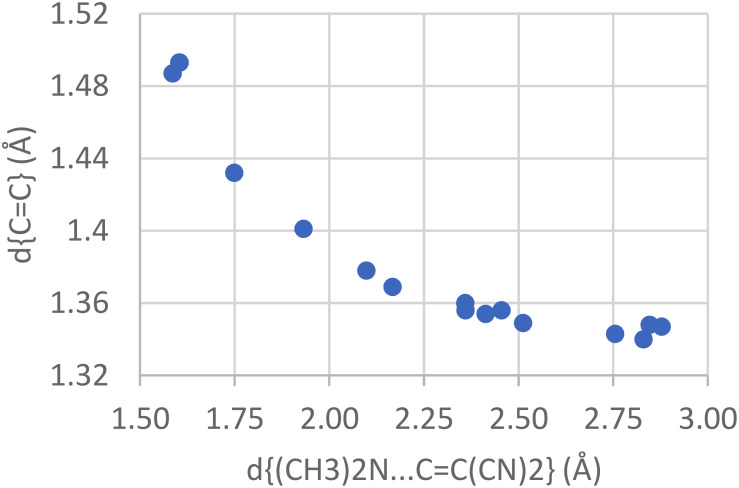
Correlation between d(N…C) and d(C = C) in 1,8-disubstituted naphthalene and 2,2′-disubstituted biphenyl compounds carrying (CH_3_)_2_N and C = C(CN)_2_ groups. [Data from CSD, Bristow *et al.*, 2020; J. O'Leary and J. D. Wallis, (2009).]

**FIG. 6.**
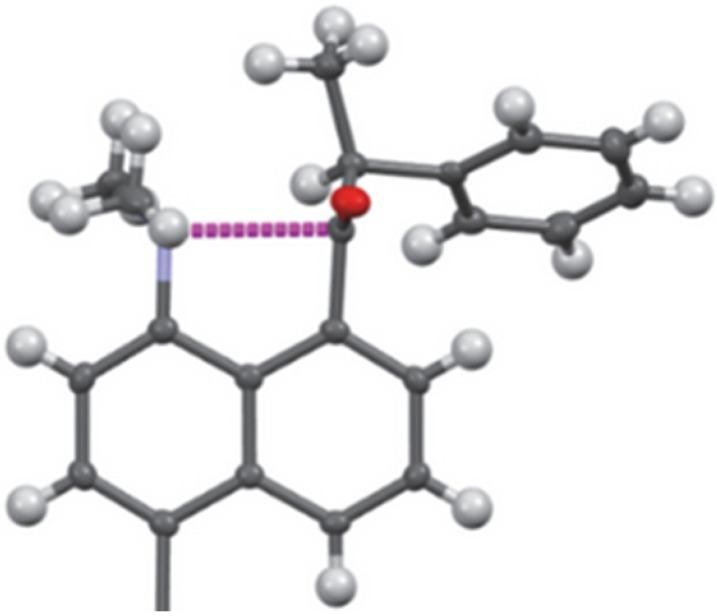
Model of an incipient stage of a Felkin–Anh controlled nucleophile addition (Ph-group in position 4 omitted. Adapted from Bristow *et al.* (2023)).

Over the years, the nucleophile–electrophile interaction was studied with peri-substituted naphthalene derivatives carrying nitrogen or oxygen nucleophiles in position 1 and various carbon electrophiles in position 8, e.g., carbonyl groups, enones, cyanides, and alkynes. As an example, Fig. 5 shows the correlated changes between N…C and C=C distances in naphthalene (and biphenyl) compounds substituted with (CH_3_)_2_N in 1(2)- and C=C(CN)_2_ in 8(2′)-positions (see Fig. 6 for a related compound). As for the I.I.I and O.H.O examples, the correlation extends continuously from typical bond distances to distances approaching van der Waals values. The following paragraphs summarize some developments based on or related to such interactions of variable strength.

### A. Mapping the charge density during N–C bond formation

Rees *et al.* (2021) have measured the electron density distributions in peri-substituted naphthalene molecules with an N(CH_3_)_2_ group in position 1 and a range of HC=CR_2_ electrophiles in position 8 showing N…C distances between ∼2.7 and 1.6 Å. With decreasing distance, the electron density ρ at the bond critical point of the emerging C–N bond increases as expected (from 0.13 to 1.35 eÅ^−3^); the Laplacian (the trace of the second derivative matrix of ρ, δ^2^ρ/(δx_i_ δx_j_)) is small and positive, ∼+2 eÅ^−5^ between 2.7 and 2 Å, i.e., typical for “non-bonded” interactions; it decreases rapidly to ∼−7 e/Å^5^ below 2 Å, thus indicating the formation of the covalent bond.

### B. Stereochemical control

An RR′C=O fragment with R ≠ R′ is prochiral. A nucleophile Nu can approach C from either side of the plane to produce enantiomeric tetrahedral molecules (RR′)NuCO. This opens the possibility of stereochemical control by hampering the approach on one side. Experimentally, it is observed that if R' is a chiral substituent, the diastereomers resulting from nucleophilic addition are formed in unequal amounts, implying that attacks from opposite sides occur with different rates. This observation was interpreted in terms of steric and electronic factors and validated by quantum chemical arguments calculated for H^−^ + HR^*^C=O in the mid 70s (R^*^=C(H Cl CH_3_)). At the time, such calculations were major undertakings, even with STO-3G minimal basis sets. The chemical and computational results are summarized in the Felkin–Anh rules (Anh and Eisenstein, 1977; Houk *et al.,* 1986), which make explicit reference to the Nu...C=O approach angle of 100–110°. Crystal structures of peri-substituted naphthalenes with nucleophiles in position 1 and a chiral substituent R^*^C=O in position 8 (e.g., H, CH_3_ and C_6_H_5_ or C_2_H_5_ at the stereogenic carbon atom, next to the carbonyl group) represent models of the early stages of Felkin–Anh situations (Fig. 6; Bristow *et al.,* 2023). Note that R^*^ in R^*^C=O chooses a conformation exposing the less hindered side to N(CH_3_)_2_.

### C. Decay of tetrahedral intermediates, estimating transition state structures and energies

**FIG. 7.**
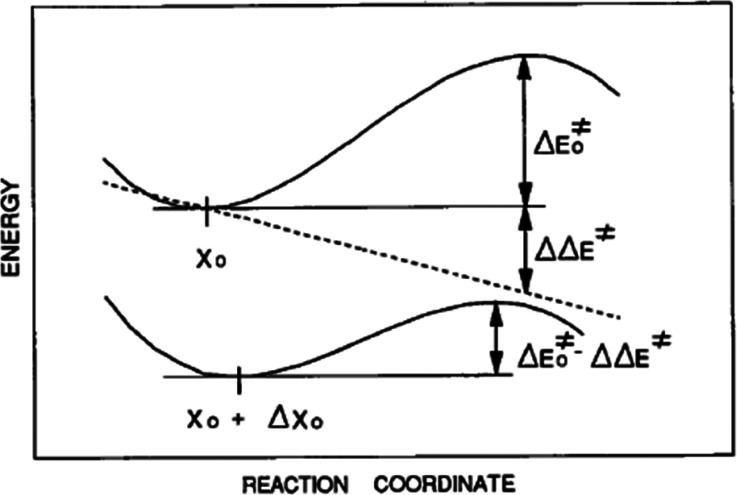
(Top) Reaction profile for the spontaneous C–O cleavage of a tetrahydropyranyl acetal; (bottom) effect of a linear perturbation (dashed line, top) simulating a better RO^−^ leaving group. (Reproduced by permission from Bürgi, Perspectives in Coordination Chemistry. Copyright 1992 by The Wiley-VHCA AG, Zurich, Switzerland.)

Tetrahedral intermediates are endpoints of nucleophilic addition reactions and starting points of the reverse cleavage reactions (see formula). Leaving groups RO^−^ with different electronic properties and different lengths of the cleavable RO–C bond may be considered as incipient stages of the cleavage reaction. For a collection of eight tetrahydropyranyl acetals undergoing spontaneous cleavage, crystal structures were combined with force constants describing molecular deformations and with free energies of activation (obtained from a linear free energy relationship; Kirby, 1994). The activation energies show a steep dependence on the lengths of the cleavable C–O bond. The difference in the length of the shortest to the longest cleavable C–O bonds is ∼0.07 Å and is accompanied by a steep decrease in activation energy from ∼39 kcal mol^−1^ (for RO^−^ = alkoxide) to ∼17 kcal mol^−1^ (for RO^−^ = 3,5-dinitrobenzoate) implying δE^‡^/δd(C–O) ∼30 Kcal {mol 0.1 Å}^−1^!). This behavior could be simulated quantitatively by a set of reaction profiles expressed as the sum of a single, constant third order polynomial in the (2D) reaction coordinate, and a variable linear term, which models the changes in the nature of the leaving groups (Fig. 7). A better leaving group has a longer cleavable C-O bond and shows better stabilization of the developing negative charge and a lower activation energy (Bürgi and Dubler-Steudle, 1988). A lengthening of the C–O bond by 0.03 Å reduces the activation energy for bond cleavage by ∼9 kcal mol^−1^ corresponding to a rate increase by six orders of magnitude, typical of enzymatic acceleration. The model also predicts a transition state structure with ∼1.95 Å for the C...^−^OR bond and ∼1.27 Å for the C=O^+^ bond in the ring. Note that the former value is very similar to the value at which the Laplacian discussed above starts indicating the formation of the covalent bond during the reverse reaction.


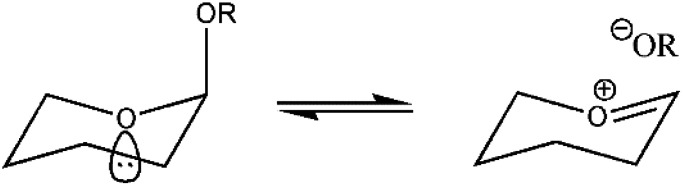


### D. Nu…C=O interactions in proteins and elsewhere

The initial stages of nucleophilic attack to C=O fragments have been characterized from small molecule crystal structures for a wide range of nucleophiles with the electrophilic C=O groups of RR′C=O, RC=OOR′ or RC=ONHR′ (Bürgi, 2002; Paulini *et al.,* 2005). Such Nu…C=O interactions are sometimes viewed in terms of a nitrogen or oxygen lone pair *n* interacting with the antibonding π orbital of the C=O group and are therefore often referred to as *n* → π^*^ interactions. Here, we review two examples of such interactions, of importance in the context of protein structure and function.

### E. C=O…C=O interactions and secondary protein structures

Fufezan (2010) retrieved a non-redundant set of ∼5300 protein structures encompassing ∼1.2 × 10^6^ residues from the Protein Data Bank (PDB), the sister product of the CSD. Of these, ∼0.77 × 10^6^ showed a nucleophilic O within 3.3 Å of the carbonyl group. The overwhelming majority of these cases, namely, 0.55 × 10^6^ or 45% of all residues, showed an O…C contact of C=O in residue *i* − 1 with C=O in residue *i*; >98% of these are found in secondary helical structure motifs (in addition to the =O_i_…H–N_*i*__+ 4_ hydrogen bonds stabilizing α-helices). These interactions, named *n_i_*_−1_→π_i_^*^ interactions, were interpreted as stabilizing these motifs (relative to alternative protein conformations). The structure directing importance of *n* → π^*^ interactions has also become evident from many conformational studies of proline derivatives, peptides rich in proline and peptoids, all of which lack H at the peptide N and can thus not form the structure directing N–H…O=C hydrogen bonds in α-helices (Wilhelm *et al.*, 2014; Newberry and Raines, 2017).

### F. Processing non-L-α-amino acids by ribosomes

Efforts to expand the genetic code while maintaining the ribosomal protein synthesis machinery raise the question of efficient N–C bond formation for non-canonical amino acids. Watson *et al.* (2023) combined the results of three experiments: (1) an electron diffraction structure of the Escherichia coli ribosome carrying methionine monomers attached to full-length tRNA; (2) metadynamic simulations of free energies surfaces (FES) founded on the experimental structure; and 3) the efficiency of the incorporation of non-canonical amino acids: aminobenzoic acid derivatives, β^3^-amino acids, and β^2,3^-cyclic amino acids. They analyzed the calculated FES and concluded: “Minima in these FESs clearly differentiate reactive and non-reactive monomers: reactive monomers across all structural classes populate a conformational space characterized by an A-site nucleophile to P-site carbonyl distance (N_α_–C_sp2_ distance) of <4 Å and a Bürgi–Dunitz angle (α_BD_) of 76–115°. Monomers whose free energy minima lie outside a region in which the N_α_–C_sp2_ distance is less than 4 Å, even with an acceptable Bürgi–Dunitz angle, do not react efficiently.”

## IV. REACTION PATH MAPPING, INCIPIENT CHEMICAL REACTIONS, SECONDARY BONDING, AND SOME GENERALIZATIONS

The correlations presented so far can be interpreted from different viewpoints. The continuous nature of the distributions over large ranges of structural parameters suggest an interpretation in terms of mappings of chemical reaction path, e.g., associative ligand exchange, nucleophilic substitution (S_N_2), proton exchange, or nucleophilic addition as discussed above. The areas near the centers of the distributions provide models of possible transition state structures. Their tails indicate incipient stages of chemical reactions (Rosenfield *et al.,* 1977).

When emphasizing the viewpoint of chemical bonding, the correlations found for (I.I.I)^−^ and O.H.O fragments are both examples of three-center-four-electron (3c-4e) systems, thus explaining the similarity of the respective correlation plots. The relevant (unnormalized) occupied molecular orbitals in the center of the (I.I.I)^−^ distribution are {5*p_z_*(I_1_) − *λ* 5*p_z_*(I_2_) + 5*p_z_*(I_3_)} (bonding) and {5*p_z_*(I_1_) − *5p_z_*(I_3_)} (nonbonding) with two equal I.I distances. In the tails of the distribution, the relevant orbitals are approximately *σ*(I_1_ − I_2_) and {λ′ σ^*^(I_1_ − I_2_) + 5*p_z_*(I_3_)} with a short and a long I.I distance. The former is usually considered a normal covalent, sometimes polar (primary) bond. The latter has been called a *secondary bond* (Alcock, 1972; here secondary *halogen* bond) and is often substantially smaller than the sum of the van der Waals radii but longer than the sum of the covalent radii. For a (symmetric) O.H.O group, the corresponding orbitals are {2*p_z_*(O_1_) + *λ″* 1*s*(H) − 2*p_z_*(O_2_)} (bonding) and {2*p_z_*(O_1_) + 2*p_z_*(O_2_)} (nonbonding) with two equal O.H distances. For an (asymmetric) hydrogen bonds, it is approximately *σ*(O_1_–H) and {λ‴ σ^*^(O_1_–H) + 2*p_z_*(O_2_)} with a short O–H bond and a long H…O distance, usually less than the sum of the van der Waals radii (hydrogen bond). For both examples, the magnitudes of the mixing coefficients λ, λ′, etc. depend on the geometry and electronic environment of the atoms forming the 3c-4e fragments X.E.X. The energies of the primary and secondary bonds are usually a sum of covalent (charge transfer), electrostatic (including polarization), and dispersion contributions (de Azevedo Santos *et al.,* 2023). The relative importance of these energies depends on the nature of X and E and the position of a given system in the overall distribution and thus the environment they are embedded in.

The secondary or hydrogen bonding characteristics of the tails of the distributions are often referred to as n → σ^*^ interactions to emphasize the partial covalent character of the interaction or as *σ* hole interaction, which emphasizes the electrostatic complementarity between the negative lone pair *n* and the positive area of the molecular electrostatic potential near the location of the σ^*^ orbital. Crabtree has written a very readable tutorial reviewing this concept (Crabtree, 2017).

Nucleophilic addition N…C=O can be discussed in an analogous way. For an incipient addition, the relevant orbitals are, approximately, *π*(C=O) and {*n*(N) + λ”” π^*^(C=O)} with a short C=O bond and a long, incipient N…C contact. Again, three centers and four electrons are involved, albeit in a bent arrangement. This situation is sometimes referred to as *n* → π^*^ or *π* hole interaction. Related examples include donor–acceptor complexes of BF_3_ and SO_3_ mentioned in Sec. V.

**FIG. 8.**
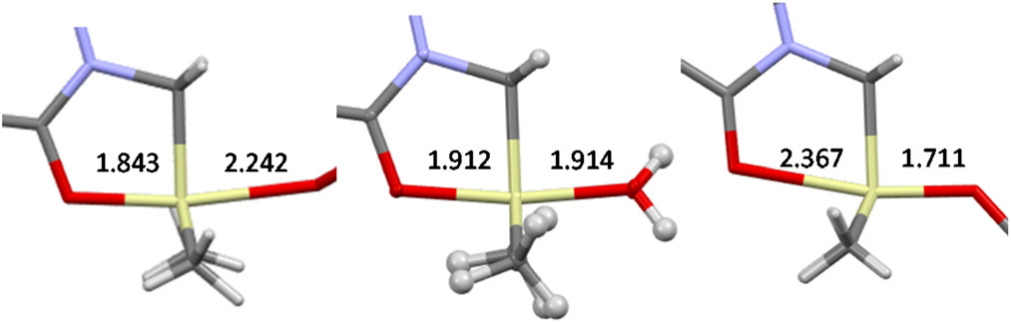
Three snapshots of an O.SiR_3_.OR' fragment along the S_N_2 path showing Si.O distances (Å; HOR' = triflic acid, pK_a_∼−15; H_3_O^+^, pK_a_∼0; phenol, pK_a_∼10; CSD entries PENDUH, KEKSOK, WENBIA).

The simple 3c-4e bonding picture lends itself to generalization. (Nearly) linear X.E.Y fragments have been observed for E = CR_3_, SiR_3_, GeR_3_, Sn R_3_ with a wide variety of nonmetal Xs and Ys. The resulting fragments may be symmetric or asymmetric. The d(X.E), d(E.Y) and α(X.ER) angle correlations are reminiscent of S_N_2 reactions (Walden inversion). Figure 8 shows three snapshots taken at the beginning, the middle, and the end of the path for an O.SiR_3_.OR' fragment. The position along the path correlates with the pK_a_ of the incoming nucleophile ^–^OR′ in analogy to the observation described for hydrogen bonds above. Secondary bonding situations observed in these species are referred to as tetrel bonding.

Analogous secondary bonding with or without charge assistance is observed for E = P, S, Cl, their relatives in subsequent rows of the periodic system and some noble gases. Current names for these interactions are pnictogen, chalcogen, halogen, and noble gas (secondary) bonding. Secondary bonding of Lewis bases to trigonal planar Lewis acidic groups, such as C=O(OR), C=O(NR_2_), C=NR, C≡N, BR_3_, AlCl_3_, SO_3_, etc. have been called triel bonding (Grabowski, 2020b, see also Sec. V).

Much of the early work on continuous correlations and secondary bonding situations pertaining to 3c-4e systems has been documented up to 50 years ago in the primary literature and summarized in books (Bent, 1968; Alcock, 1972; Bürgi, 1975; and Dunitz, 1979; 1995; and Bürgi and Dunitz, 1994) Since then, the number of studies on secondary bonding has surged. A useful compilation of review articles published since 2013 is found in a virtual special issue of Acta Crystallographica (Brammer *et al.,* 2023). Secondary bonding in noble gas compounds has also been reviewed (Bauzá and Frontera, 2020).

## V. WHEN IS A BOND (NOT) A BOND? (Alvarez *et al.,* 2009)

The correlations described above show continuous transitions from short interatomic distances, “bonds,” to long distances often characterized as “non-bonded.” The former are usually associated with covalent or ionic bonds, the latter with so-called “non-covalent” or “non-bonded” interactions. For the latter, the term “secondary bond” (Alcock, 1972) seems more appropriate provided they are shorter than the sum of van der Waals radii. This choice does not exclude covalency (viz., “non-covalent”) nor does it imply the absence of bonding (viz., “non-bonded”).

The *continuous* nature of the correlation curves may sometimes prevent an unambiguous separation between bonds and secondary bonds. A convenient, qualitative way out of such ambiguities for an X.E.X fragment is to label the shorter of the two X.E distance as the (primary) bond and the longer one as the secondary bond. For an X.E.Y fragment, the distance closer to the sum of the respective covalent radii defines the bond, the other distance implies a secondary bond. In general, there is a tendency for the more nucleophilic terminal atom to form the (primary) bond. Proton affinities or negative pK_a_ values may serve as rough proxies of nucleophilicity in these Lewis acid/Lewis base interactions (see the discussion of hydrogen bonds in Sec. II, the O.SiR_3_.O fragments in Sec. IV and the O.ML_4_.O fragments in Sec. VI).

For a more quantitative characterization, the energy for a given interatomic interaction can be decomposed into different contributions: quantum mechanical orbital overlap between the two interacting fragments (including covalent bond formation, charge transfer, and polarization), electrostatic energy between the two fragments, Pauli repulsion between the filled orbitals of the two fragments, and dispersion interactions (Vermeeren *et al.,* 2020). Covalency may dominate at short internuclear distances, electrostatics and dispersion at long ones, but their relative importance depends on the atoms involved and the specific geometrical situation. Consider a quantum chemical calculation on the nucleophilic attack of cyanide ion to acetone, NC^−^ + (CH_3_)_2_C=O, as an example. It shows that the orbital overlap contribution is comparable to the electrostatic term long before NC^−^ reaches the estimated transition state at d(NC…C) ∼ 1.98 Å (Ríguez *et al.*, 2023; Fernández *et al.*, 2023; compare with the experimental estimate given in the paragraph on “Decay of tetrahedral intermediates” in Sec. III.C).

The dominating contributions to an interatomic interaction depend not only on the nature of the atoms X, Y, and E involved in a bond/secondary bond situation and on the atoms directly attached to X and Y but also on the wider environment of the fragment. Extreme examples of the influence of the environment on molecular geometry and the nature of the corresponding bonds or secondary bonds are provided by some donor–acceptor complexes. In the gas phase, the B.N distance in CH_3_CN-BF_3_ is 2.01 Å; in the crystal, it shrinks by 0.4 Å to 1.63 Å; corresponding N.S distances in H_3_N–SO_3_ are 1.96 and 1.77 Å. In these and related examples, the distinction between bond and secondary bond is entirely arbitrary. As the N.B and N.S distances shorten the angles N–B–F and N–S–O increase from ∼96° toward 109.5° (Triel secondary bonding or *n*(N) → π^*^(BF_3_ or SO_3_) interaction). The substantial deformations from the gas phase to the solid state structures require reorganization energy, which is overcompensated, however, by stabilizing lattice energies. The correlated distance/angle changes are reminiscent of the reaction path for S_N_1 substitution reactions at trigonal centers (Burns *et al.,* 1999; Murray-Rust *et al.,* 1975).

The above consideration also applies to structure correlations if no gas phase geometry is available for comparison. The magnitude of environmental effects has been estimated along the following lines: the tables of reference distances in volume C of the International Tables of Crystallography also provide “standard deviations σ” (Prince, 2006). These numbers reflect three effects

$$\sigma^{2}={\;\sigma }_{\text{intra}}^{2}+\;\sigma_{\text{packing}}^{2}+\;\sigma_{\;\text{exp}\;}^{2}.$$

σ_intra_ accounts for intramolecular differences in A and/or B in an AX–YB fragment of interest. Their magnitude is nearly equal to the overall σ. In contrast, σ_packing_ has been estimated from molecules with identical connectivity but different packings, e.g., from polymorphs, from structures with more than one molecule in the asymmetric unit, from different solvates or from series of salts with one ion the same, while the other varies. Finally, σ_exp_ accounts for experimental uncertainty estimated by least squares refinement of the diffraction data. Order of magnitude estimates of these contributions have been presented for interatomic distances, angle, and torsion angles in some metal complexes (Martin and Orpen, 1996). The σ_packing_ are found to be clearly larger than the experimental uncertainties and – although the differences are not as large as the ones for the BF_3_ and SO_3_ adducts – they must be largely assigned to packing effects.

Given a freely chosen reference member of a population with its environment, its neighbors in the distribution will have different environments, the difference acting as a perturbation on the PES of the reference member and shifting it to a new position in the population. For a given strength of the perturbation, such shifts are large in directions in which the reference PES changes slowly and small if the PES increases steeply.

**FIG. 9.**
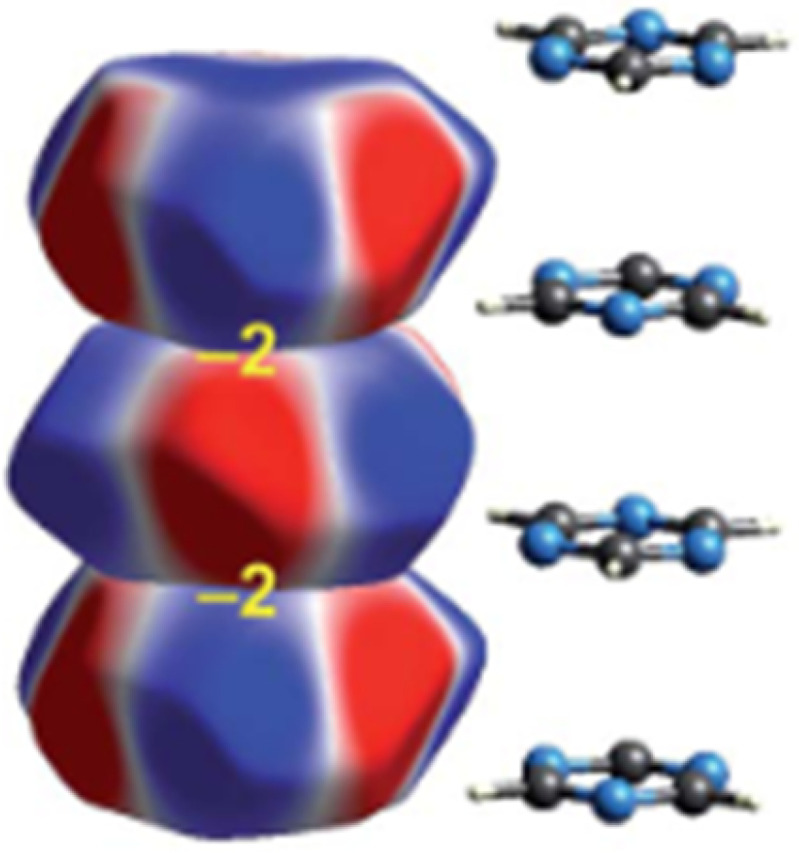
Electrostatic complementarity in stacks of crystalline s-triazine. Electrostatic energy between nearest neighbors estimated at −2 kJ mol^−1^. (Reproduced by permission from Edwards *et al.*, Faraday Discuss. **203**, 93 (2017). Copyright 2017 by The Royal Society of Chemistry.)

These qualitative observations have several qualitative consequences. They imply that:

• the shapes of structure correlations help to distinguish between low and high energy regions of a PES but do not provide quantitative information (principle of structural correlation, Murray-Rust *et al.,* 1975);
• chemical (primary and secondary) bonds, e.g. X-E and E…Y in X-E…Y are not only determined by the nature of the bonded atoms X, E, and Y but also – sometimes to a larger and sometimes to a smaller extent - by their environment, substituents on X and Y as well as solvent shells or crystal packings;
• Sample points representing X.E.Y fragments with similar X and Y in equal or comparable environments tend to be found in the middle of a distribution. For unequal environments, sample points congregate in the tails of the distributions;
• last, but not least: although secondary bonds may sometimes be strong enough to be structure determining or to exhibit a high probability of occurrence, other interactions such as the global intermolecular electrostatic or dispersion interactions may well be as important as the local secondary bonding (Fig. 9; Edwards *et al.,* 2017; Alvarez, 2013; and Spackman *et al.,* 2023).

## VI. OTHER STRUCTURE CORRELATIONS, LIGAND EXCHANGE AT METAL IONS, CLUSTER REARRANGEMENT

### A. Some early, prototypical structure correlations involving metal atoms

**FIG. 10.**
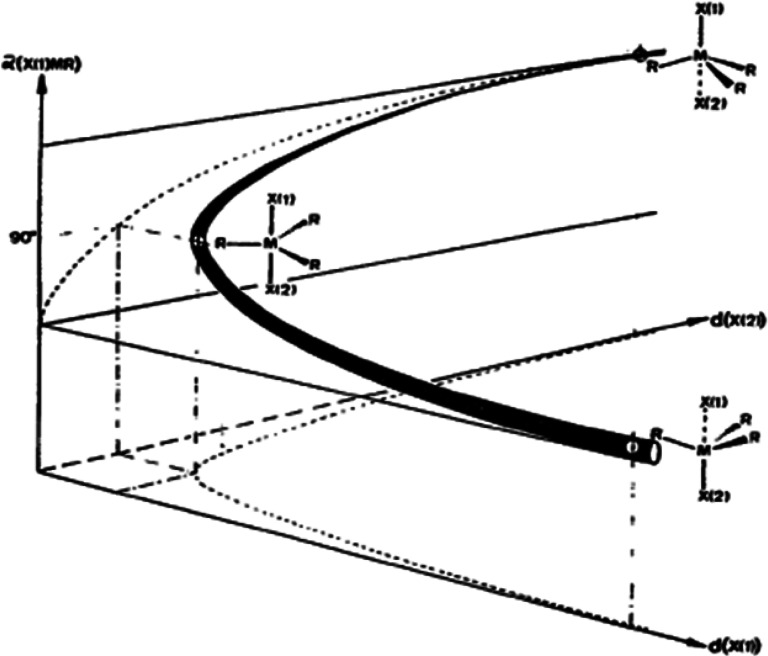
Correlated changes of d(M.X1), d(M.X2) and α(X1.M.R) (solid line) with projections on to the d(M.X1)/d(M.X2) and d(M.X1)/α(X1.M.R) planes (dotted lines; M = Cd, R = SR. (Reproduced by permission from H. B. Bürgi and J. D. Dunitz, Acc. Chem. Res. **16**, 153–161 (1983). Copyright 1983 by The American Chemical Society).

Structure correlations are not limited to interatomic distances nor to fragments containing main group elements. As can be seen in Fig. 10 above for a O.SiR_3_.O fragment, the lengthening of O–Si correlates not only with a shortening of Si…O; it is also closely followed by a decrease (increase) of the O–Si–R (R–Si…O) angles from ∼110° to 90° to ∼70° (∼70° to 90° to ∼110°) through a five coordinate, intermediate trigonal bipyramidal structure. The first such trajectory was mapped with complexes of Cd^2+^, a 4d^10^ transition metal ion coordinated to various electronegative ligands L, L′ and thiolates ^–^SR:

$$L-\text{Cd}{\left(\text{SR}\right)}_{3}\ldots L'\;\to \;L-\text{Cd}{\left(\text{SR}\right)}_{3}-L'\;\to \;L\ldots {\left(\text{SR}\right)}_{3}\text{Cd}-L'.$$

(Fig. 10; Bürgi, 1973). This behavior is not surprising since the L.Cd.L' fragment can also be considered as a 3c-4e system with L and L' contributing a lone pair of electrons each, whereas the filled 4d^10^ shell of Cd^+2^ contributes little to the Cd.L and L' bonding. In the parlance of organic chemistry, Fig. 10 maps an S_N_2 pathway; in inorganic chemistry, the correlation is considered to map the pathway of associative ligand exchange at a tetrahedral metal complex.

**FIG. 11.**
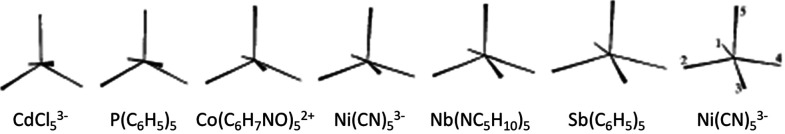
First mapping of the Berry mechanism. (Reprinted with permission from Muetterties & Guggenberger 1974. Copyright 1974 American Chemical Society)

Muetterties and Guggenberger (1974) combined an astonishing diversity of structural fragments to map the transformation of a trigonal bipyramid to a square pyramid, i.e., the Berry mechanism (Fig. 11). A similar correlation was established with a variety of five coordinate phosphorus compounds (Holmes, 1984). These correlations suggest that the NMR equivalence of fluorine atoms in the related trigonal bipyramidal molecule PF_5_ is due to an axial/equatorial exchange of fluorine atoms via a Berry mechanism.

**FIG. 12.**
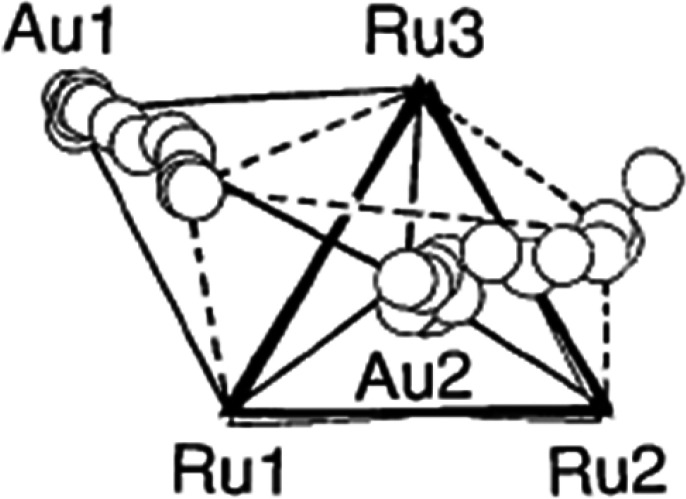
Berry mechanism rationalizing exchange of the axial Au1 and Ru2 with the equatorial Au2 and Ru1 in a trigonal bipyramidal Au_2_Ru_3_ cluster (solid lines) via a square pyramid (dashed lines. Reprinted with permission from A. G. Orpen and I. D. Salter, Organometallics **10**, 111–117 (1991). Copyright 1991 by the American Chemical Society).

A series of M_2_Ru_3_ clusters (M = Cu, Ag, Au) provide another example of the Berry mechanism. Correlating their structures suggests that the NMR spectroscopically observed the equivalence of the two M atoms, one in an axial and one in an equatorial position of a trigonal bipyramid, probably takes place via a square pyramid with the two M atoms in the equatorial position (Fig. 12; Orpen and Salter, 1991).

### B. Five coordinate metal complexes

**FIG. 13.**
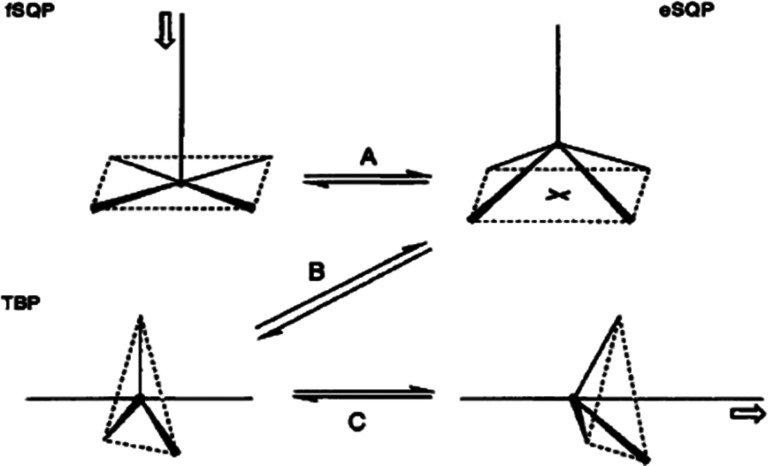
Main coordination geometries and deformation pathways of five coordinate complexes: (a) first step of associative ligand substitution, (b) Berry mechanism, and (c) associative ligand substitution. (fSQP, eSQP = flattened, elevated square pyramid; TBP = trigonal bipyramid. (Reproduced by permission from Auf der Heyde, Angew. Chem. Int. Ed. **33**, 823–839 (1994). Copyright 1994 by the Wiley).

There are many more examples of structure correlations involving various combinations of angle and distance changes. The most prevalent correlations characterizing metal five coordination are sketched in Fig. 13. Path A can be considered as the initial step of an associative ligand exchange reaction, which then might be followed by an exchange of the apical with an equatorial ligand via a (reverse) Berry mechanism B and completed by elimination A of the new apical ligand, in short fSQP → eSQP → TBP → eSQP′ → fSQP′. An analogous sequence of events can be imagined for the associative ligand exchange reaction of tetrahedral complexes: T + L → TBP → T′ + L′. These possibilities have emerged from studying several classes of five coordinate metal complexes, including Fe, Ni, Cu, Zn, Mo, Rh, Ir, Pd, and Pt complexes (Auf Der Heyde, 1994; Murphy and Hathaway, 2003). Analogous studies are available for six-, seven-, and eight-coordination (Muetterties and Guggenberger, 1974; Klebe, 1990; 1994; and Yao *et al.,* 2001). These studies have confirmed earlier guesses on the structural changes during ligand rearrangement and substitution reactions at a time when accurate quantum chemical calculations for these many-electron system were not accessible.

### C. Structure-reactivity correlations

For a series of O≡ML_4_-OR complexes (M = Tc(V), Re(V)) with different ligands L, the M–O distance has been correlated with the pK_a_ of HOR: the more basic ^−^OR, the shorter the M–O distance. While the O≡M distance lengthens only slightly, the O≡M stretching frequency drops substantially (∼100 cm^−1^). Reaction profiles for the dissociation of ^−^OR analogous to those for the acetal cleavage discussed above (Fig. 7) have been constructed (Ferretti *et al.,* 1999). They confirm the conclusion obtained from the acetal study, namely, that a relatively small increase in the cleavable bond is indicative of a large decrease in activation energy, here δΔG^ǂ^/δd(O≡M) ∼60 kcal (mol Å)^−1^. This value corresponds to a rate increase of ca. four orders of magnitude for an increase in the length of the cleavable bond by ∼0.1 Å. The δΔG^ǂ^/δd values for different cleavable bonds range from ∼60 to ∼300 kcal (mol Å)^−1^ (Bürgi, 1992; 1998).

## VII. FLUXIONAL MOLECULES, CONFORMATIONAL INTERCONVERSIONS

Fluxional molecules are characterized by many minima on their PES with nearly equal energies and low barriers between the minima. They should be ideal candidates for structure correlations. Two examples have been mentioned above: the axial/equatorial exchange of R in PR_5_ molecules (PF_5_), of Au in Au_2_Ru_3_ clusters, and of other systems showing ligand exchange via the Berry mechanism.

Many conformational degrees of freedom involving rotation about single bonds unhindered by their environment come with low activation barriers. Correlations between two torsional degrees of freedom have been analyzed for many different X(phenyl)_2_ fragments (Klebe, 1990; 1994) and for several systems with between three and eight conformational degrees of freedom (e.g., O=P(phenyl)_3_, Bye *et al.,* 1982; M{P(phenyl)_3_}_2_, Norskov-Lauritsen and Bürgi, 1985). Three examples serve as illustrations: gearing motion of two rotors, pseudo-rotation in five membered chelate rings, and the Ramachandran φ,ψ correlation plot.

**FIG. 14.**
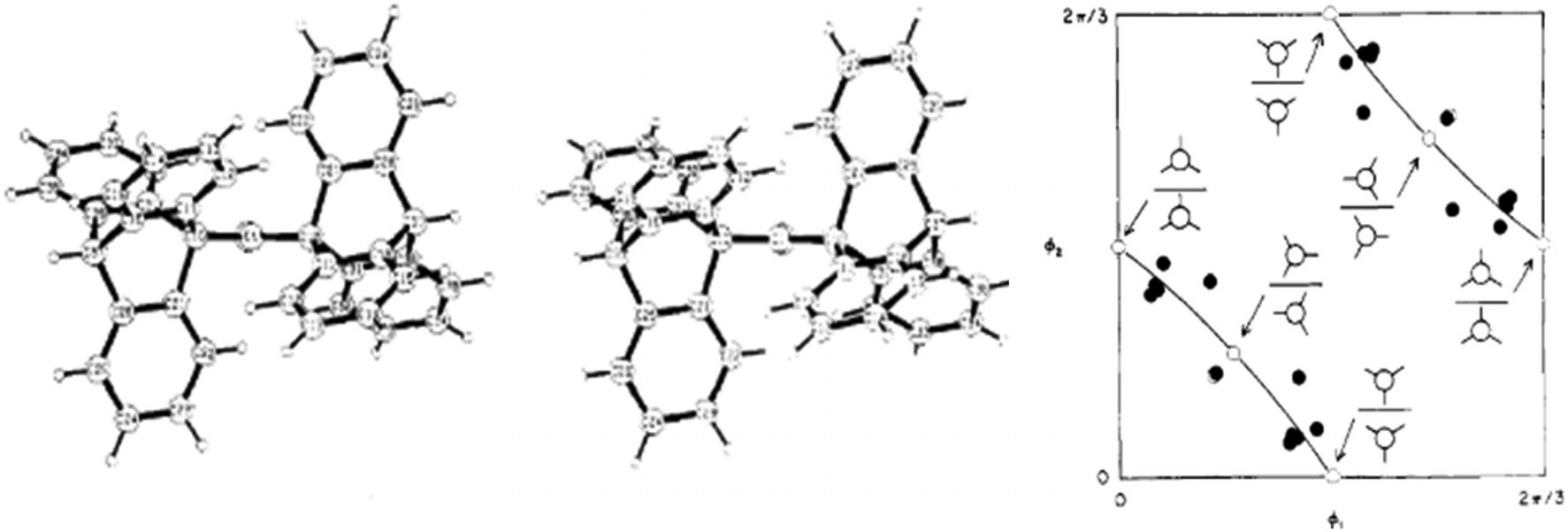
Stereo view of bis(9-triptycyl) ketone (Tp_2_C=O) viewed down the C=O direction (top). Conformational map for Tp_2_C=O, Tp_2_CHOH, and Tp_2_CH_2_ (5 independent observations, bottom). (Reprinted with permission from Johnson *et al.*, J. Am. Chem. Soc. 104, 5163–5168 (1982). Copyright 1982 by the American Chemical Society).

Figure 14 (left) shows the x-ray molecular structure of bis(9-triptycyl)ketone (Tp_2_C=O). The two triptycyl groups are tightly interlocked. The arrangement is reminiscent of geared cogwheels. The torsion angles of the two triptycyl groups for three derivatives (Tp_2_CH_2_, Tp_2_CHOH, Tp_2_C=O) are clearly correlated: as the torsion angle of one triptycyl group increases, the one of the other group decreases (Fig. 14, right). The pathway traced out in the conformational map can be interpreted as describing a gearing motion. Changes in energy along this path calculated with a force field are small, ranging from 0.2 to 1.9 kcal mol^−1^ depending on the derivative (Johnson *et al.,* 1982).

**FIG. 15.**
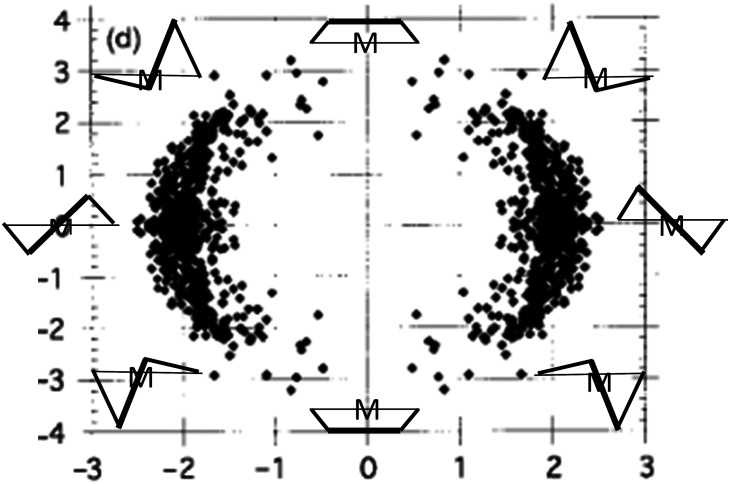
Conformational map of bis(diphenyl-phosphino)ethane from a principal component analysis. C_2_ symmetric λ and δ twist conformations (left and right), C_s_ symmetric envelope (top and bottom), and asymmetric envelops (338 independent observations). Adapted from Harris *et al.* (2001) with permission of the Royal Society of Chemistry.

Many bidentate ligands form five membered chelate rings with metal atoms. Figure 15 shows the distribution of conformations for bis(diphenylphosphino)ethane as obtained from a principal component analysis (Harris *et al.,* 2001). The first two principal components are shown in Fig. 15. They explain 99.97% of the total variance of the five torsion angles in the ring. The distribution of sample points traces a pseudo-rotation pathway. The preferred forms are the C_2_ symmetric λ and δ twist conformations. Harris *et al.* (2001) suggest that the absence of C_*S*_ symmetric envelope conformations in the plot indicates that they are energetically unfavorable and that they might be the transition state for the λ to δ interconversion.

**FIG. 16.**
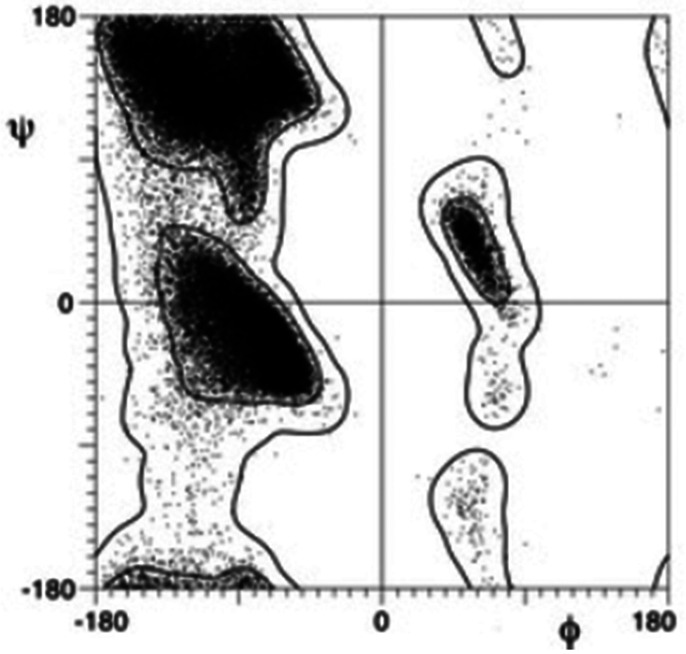
Ramachandran plot from 97 368 residues with backbone *B*-factor < 30 from the 500-structure high-resolution database. (Reproduced with permission from Lovell *et al.*, Proteins **50**, 437–450 (2003). Copyright 2003 by The Wiley).

The distribution of torsion angles in chain like molecules will influence their overall structural appearance. This has been recognized early on in protein structure characterization, long before the CSD and PDB were available. The torsion angles characterizing the single bonds **C**(i−1)–**N**(i)–**Cα**(i)–**C**(i) (φ) and **N**(i)–**Cα**(i)–**C**(i)–**N**(i + 1) (ψ) of the peptide fragment are highly clustered and correlated; the φ,ψ correlation plot, i.e., the Ramachandran plot, shows regions characteristic of α-helices, β-pleated sheets, of other polypeptide structural motifs and regions that are conspicuously empty (Fig. 16; Ramachandran *et al.,* 1963; for a more recent discussion, see: Lovell *et al.,* 2003).

The purpose of structure-based drug design is to invent molecules—anything from a natural enzyme substrate to a synthetic drug—that matches a protein active site with respect to shape and electrostatic properties but does not show excessive intramolecular strain such as high energy conformations. From the combined information in the CSD and the PDB, histograms of torsion angles can be constructed, which take into account the detailed molecular environment of the rotatable bond and whose maxima indicate low energy, desired conformations. There is a rich literature on this topic, too much to be discussed here. A representative example is Schärfer *et al.* (2013).

## VIII. GLIMPSES INTO THE FUTURE AND A CONCLUSION

### A. From analysis to prediction

**FIG. 17.**
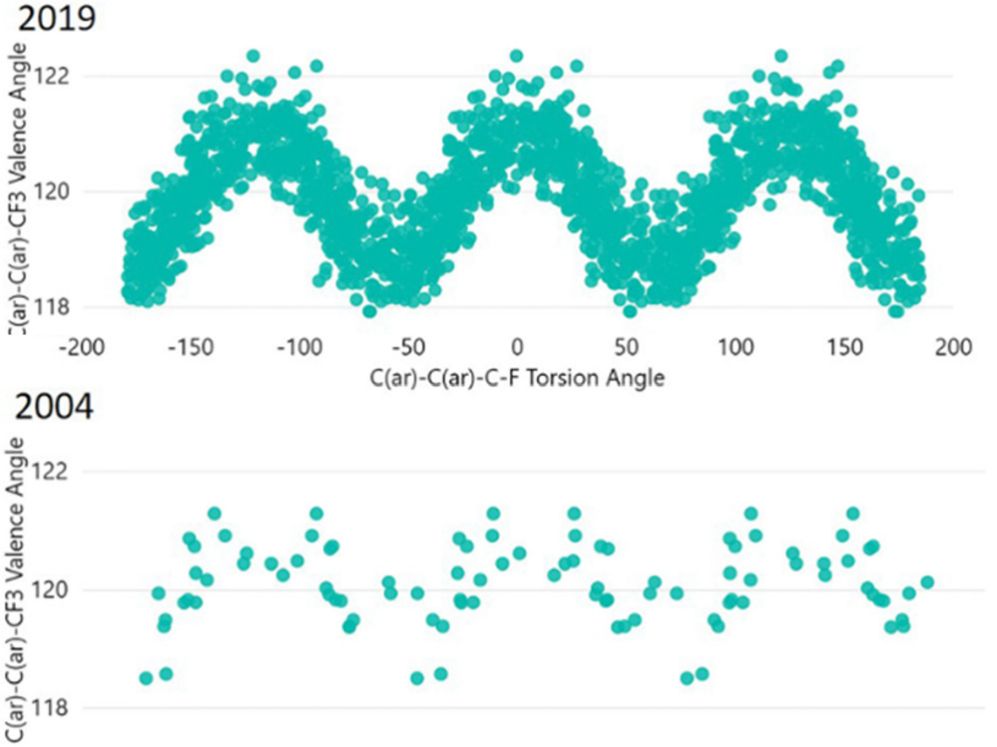
Bond angles C(Ar)–C(Ar)–CF_3_ as a function of the torsion angle C(Ar)-C(Ar)-C-F. Adapted from Cole *et al.* (2019).

So far, specific structural fragments related to specific chemical problems have been analyzed, many of them related to PES's and thus to chemical dynamics. A more general questions would be whether and to what extent the large amount of information available in the CSD can be used to predict the outcome of experiments not yet performed. A new occurrence of a known structural fragment is likely to be found inside a previously established distribution of structures. As an example, Fig. 17 compares a torsion/bond angle correlation in the aryl-CF_3_ groups from 2004 with the one in 2019 (Cole *et al.,* 2019). The earlier distribution— however sparse—already indicates the main features of the much denser 2019 distribution. Predictions of structural parameters have thus become easier and more reliable due to the continuous growth of the CSD, the increased sophistication of the CSD analysis software and—sometimes—the combination with data from other, nonstructural databases (https://www.ccdc.cam.ac.uk/media/Documentation/C0E67C8D-4F1C-4354-9F38-D562C74B4F2B/Introduction-to-ConQuest-CQ001.pdf).

A continuing problem is the prediction of crystal packings from the knowledge of the chemical constitution of a molecule. Progress in this field of research has been documented in six crystal structure prediction (CSP) competitions organized by the CCDC; a seventh competition is under way (https://www.ccdc.cam.ac.uk/community/ccdc-for-the-community/partnerships-and-initiatives/csp-blind-test/). Todays' CSPs use mostly sophisticated quantum chemical computations (e.g., Firaha *et al.,* 2023), sometimes combined with machine learning (ML) techniques. As an alternative, maybe naïve, approach, one might consider to predict crystal packing based more or less directly on the large number of observed packings stored in the CSD using machine learning. One approach is to develop an intermolecular force field expressed as a sum of pairwise, intermolecular interactions not derived from quantum chemical calculations but from as many interatomic distances as possible, between as many atom types as possible, and in as many environments as possible (Hofmann and Kuleshova, 2023). The interatomic potentials obtained from ∼259 000 structures through machine learning were scaled with 60 experimental lattice (Gibbs) energies. The scaled potentials reproduced the experimental energies with a mean error of <5.7% and densities with a mean error of 4.06%. Gibbs free energies of all the ∼259 000 compounds were calculated. Some of them turned out to be positive, suggesting that the corresponding experimental crystal structures contained errors. Scrutinizing these structures revealed missing or misplaced hydrogen atoms and other errors. This is a most useful side result of this work and a complement to the already sophisticated tests which a structure determination has to undergo before being included in the CSD.

ML methods are also used to predict (nonstructural) properties for broadly defined classes of chemical compounds. An example is the difficult problem of teaching a machine to estimate small molecule melting points from a collection of structural descriptors and to develop aqueous solubility models with these estimates. This has been achieved with a mean absolute error of ∼27 °C using the CCDC melting point dataset comprising ∼100 000 entries (Zhu *et al.,* 2023). Such estimates are important for drug development where even an approximate knowledge of these properties helps in selecting those members from a group of drug candidates, which are more or less likely to exhibit appropriate solubility and transport properties.

Another example deals with one of the attractive properties of MOFs, namely, the adsorption of guest molecules on the large inner surfaces of their cavities with applications in gas storage and gas mixture separation among others. Apart from chemical complementarity between the cavity surface and the properties of the guests, it is important that the latter can penetrate the framework. This question was rephrased: can the pore limited parameter, i.e., the diameter of the sphere diffusing through a MOF, be predicted given only the metal–linker combination. The problem is challenging because MOFs encompass a wide variety of organic linker molecules, of coordination environments, and framework topologies. Using an ML approach, guest accessibility was predicted with an ∼80% certainty (Pétuya *et al.,* 2022).

ML applications to structure related problems are expected to grow rapidly. They attempt to make optimal use of the large, computer readable amount of information in the CSD. Although ML has the potential to overcome some of the limitations of human researchers, it should be noted that we often do not know how well the space of the probable outcomes of an experiment is populated with the existing knowledge. It is therefore necessary to distinguish a prediction based on a reliable interpolation in a densely populated event space from one in a sparsely populated one or from an extrapolation at the borders of the available knowledge. If the latter is suspected, it is probably preferable to first expand the knowledge database with specific experimentation.

### B. A conclusion

This essay has tried to illustrate - with selected examples – how correlations between the distances and angles characterizing comparable chemical fragments can serve as an organizing principle of large groups of structural data, a basis for a better understanding of bonding, geometrical reaction paths, reactivity (ΔG^ǂ^), spectroscopy (IR, NMR), and other aspects of chemistry. Correlations between structures and their physical and chemical properties are more laborious to establish because databases of different properties may be differently constructed and often lack the careful selection of data which is a hallmark of the CSD. In the near future, this problem might be alleviated with artificial intelligence assistants that are able to mine information on properties from the scientific literature (Zheng *et al.,* 2023).

Some of the analyses described here are based on hundreds or thousands of individual structures and would probably not have been feasible if it were not for the availability of the computer-searchable CSD. There is much more research based in one way or another on the availability of multiple, more or less related structures in the CSD, too much to be reviewed here. The general scientific impact of the CSD has been documented with a citation analysis (Wong *et al.,* 2010). Current and recent developments are summarized on the CCDC “Discover” page (https://www.ccdc.cam.ac.uk/discover/).

The conclusion arising at the end of this review is obvious: the vast amount of work based on and emerging from the CSD turned the “passionate belief” of Kennard and Bernal into fact: collective use of data did, does, and will lead to the discovery of new knowledge!

## Data Availability

Data sharing is not applicable to this article as no new data were created or analyzed in this study.
